# Supplementary figures and images for: Genome-Wide Identification and Comprehensive Analysis of the AP2/ERF Gene Family in Pomegranate Fruit Development and Postharvest Preservation
Source: Genes (Basel). 2022 May 17;13(5):895. doi: 10.3390/genes13050895 (PMC9141937; doi:10.3390/genes13050895)

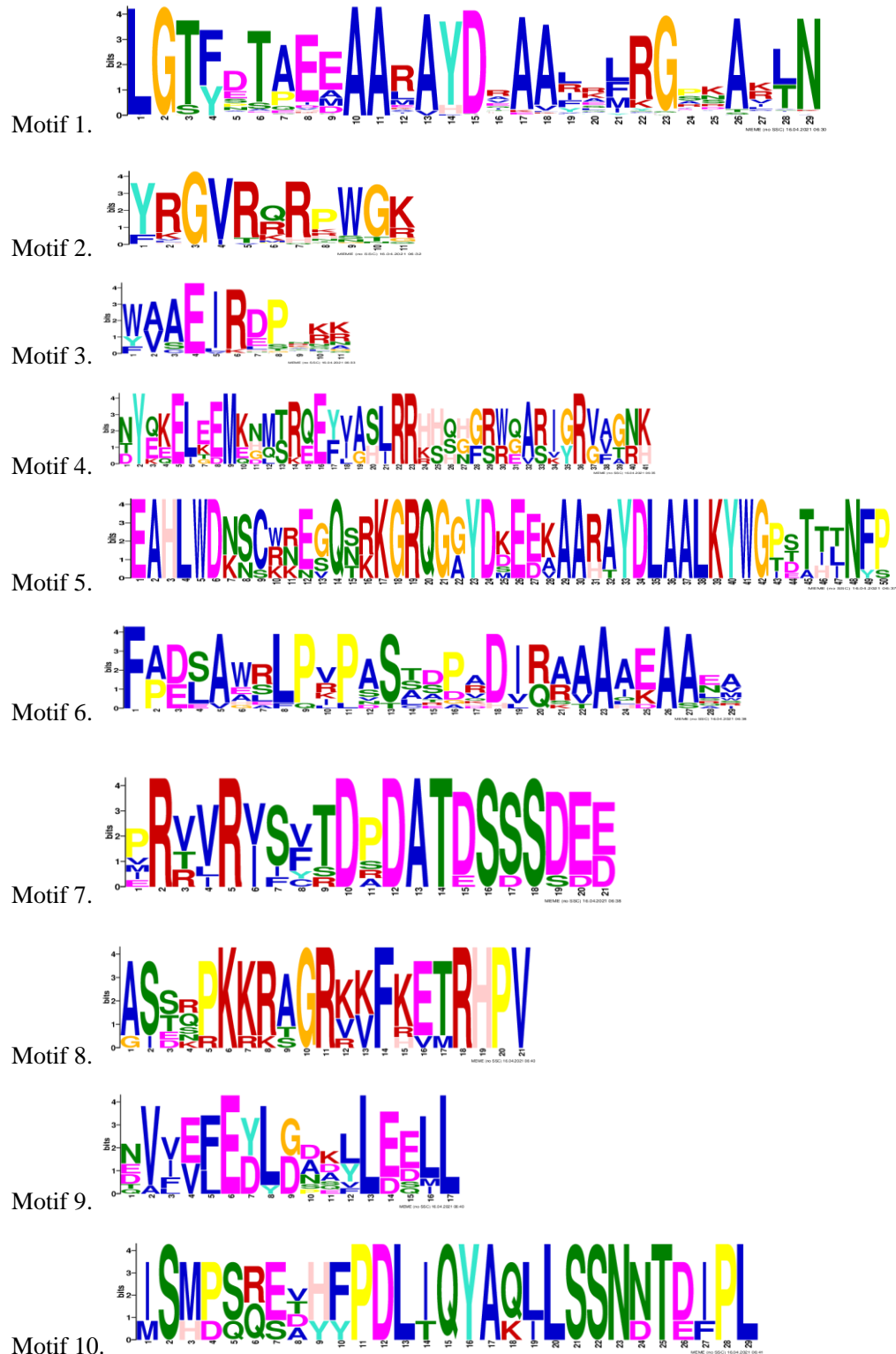

**Figure S1** The sequence information for each motif (Motif 1-Motif 10).

Supplement: Supplementary file 1 [file genes-13-00895-s001.zip › genes-1706806-supplementary/Supplementary material/Figure S1.pdf]
